# The conformations and basal conformational dynamics of translocation factor SecDF vary with translocon SecYEG interaction

**DOI:** 10.1016/j.jbc.2022.102412

**Published:** 2022-08-22

**Authors:** D.R. Weaver, D.N. Amin, G.M. King

**Affiliations:** 1 Department of Physics and Astronomy, University of Missouri, Columbia, Missouri, USA; 2 Department of Biochemistry, University of Missouri, Columbia, Missouri, USA

**Keywords:** atomic force microscopy, secretion, *Escherichia coli*, kymograph, membrane protein, protein complex, AFM, atomic force microscopy, BIC, Bayesian information criterion, DBM, dodecyl-β-maltoside, PDB, Protein Data Bank, PMF, proton motive force, Sec, secretory, SecYEG·DF, co-assembled proteoliposomes containing SecYEG and SecDF, STaSI, Step Transition and State Identification, TM, transmembrane

## Abstract

The general secretory, or Sec, system is a primary protein export pathway from the cytosol of *Escherichia coli* and all eubacteria. Integral membrane protein complex SecDF is a translocation factor that enhances polypeptide secretion, which is driven by the Sec translocase, consisting of translocon SecYEG and ATPase SecA. SecDF is thought to utilize a proton gradient to effectively pull precursor proteins from the cytoplasm into the periplasm. Working models have been developed to describe the structure and function of SecDF, but important mechanistic questions remain unanswered. Atomic force microscopy (AFM) is a powerful technique for studying the dynamics of single-molecule systems including membrane proteins in near-native conditions. The sharp tip of the AFM provides direct access to membrane-external protein conformations. Here, we acquired AFM images and kymographs (∼100 ms resolution) to visualize SecDF protrusions in near-native supported lipid bilayers and compared the experimental data to simulated AFM images based on static structures. When studied in isolation, SecDF exhibited a stable and compact conformation close to the lipid bilayer surface, indicative of a resting state. Interestingly, upon SecYEG introduction, we observed changes in both SecDF conformation and conformational dynamics. The population of periplasmic protrusions corresponding to an intermediate form of SecDF, which is thought to be active in precursor protein handling, increased more than ninefold. In conjunction, our dynamics measurements revealed an enhancement in the transition rate between distinct SecDF conformations when the translocon was present. Together, this work provides a novel vista of basal-level SecDF conformational dynamics in near-native conditions.

The export of newly synthesized polypeptide chains across membranes is a fundamental activity in cells. More than 30% of proteins in any organism are subject to this process (1, 2). Several distinct pathways have evolved to accomplish protein maneuvering across membranes, but only the general secretory (Sec) system is found in all domains of life (3, 4, 5). In *Escherichia coli*, the Sec system utilizes a translocase consisting of integral membrane translocon SecYEG in complex with peripheral SecA, an ATPase. The manner in which SecA uses ATP binding and hydrolysis to transport polypeptide chains through the protein-conducting channel in SecYEG is only superficially understood (1, 2, 6).

SecDF, a heterodimeric integral membrane protein, is a factor in the Sec translocation process that stimulates the work of the translocase (7, 8, 9). Along with translocon SecYEG and insertase YidC, SecDF is a central component of the holotranslocon complex (10, 11). There has been much speculation about the mechanism of SecDF and its interactions in the membrane. Structural and functional analyses have suggested that the large periplasmic P1 domain of SecD plays a critical role in stimulating precursor protein transport in a manner dependent on proton motive force (PMF) (12, 13, 14). Though SecDF comes into close proximity to the translocon and is thought to position its periplasmic domain over the precursor exit site of SecYEG (10, 15, 16, 17, 18), studies have suggested that YidC mediates the binding between SecDF and SecYEG (5, 19).

Recent progress in understanding the molecular processes of SecDF come from high-resolution structures in different conformations. The periplasmic domain consists of three regions: P1-head and P1-base (SecD), and P4 (SecF). Mutagenesis studies have shown that the P1-head domain is critical in the translocation process (13, 20). Structures of SecDF showcase three distinct forms that vary primarily with the conformation of P1: the super membrane facing (super F), membrane facing (F), and intermediate (I) forms. The three structural forms are thought to be directly related to SecDF function. The transmembrane (TM) region of SecDF consists of 12 helices, with TM1–6 and TM7–12 corresponding to SecD and SecF, respectively. While the super-F and F forms of SecDF show that this TM region is sealed, analysis of I form structures has revealed a channel comprising TM4, TM5, TM6, and TM10 that provides a continuous pathway from the cytoplasm to the periplasm (21). All-atom molecular dynamics simulations have revealed that the deprotonated state of Asp365 within TM 5 is likely involved with channel formation. This highly conserved residue for proton transport and protein translocation could attract water molecules from the cytoplasmic side of the membrane. It is also interesting to note that the distance between the proton-interacting TM region and the presumed precursor interaction area of the periplasmic region is large, indicative of long-range allosteric control. Researchers have shown that changes in the TM region can produce drastic structural changes in the periplasm, in particular, converting a β-sheet (present in both the F and I forms) to a β-barrel (super F form) (22).

While the aforementioned studies have produced vital information for the field, most structural analyses only provide still frames (13, 23). Molecular dynamics simulations can be used to predict and visualize membrane protein dynamics. In the case of SecDF, such simulations have revealed changes in P1-head conformations (24). Nevertheless, direct experimental visualization of SecDF protrusion dynamics in membrane is lacking. Atomic force microscopy (AFM) has become an increasingly important tool in biophysics. The method is able to image large membrane protein complexes in physiologically relevant conditions (25, 26). Our laboratory has employed AFM to analyze the dynamics of SecYEG and SecA, achieving molecular-scale (∼10 Å) lateral resolution coupled with ∼1 Å vertical resolution. In addition to imaging, kymographs or trace/retrace line scan analysis can also be used to achieve higher temporal resolution (<100 ms) (27, 28, 29).

Here, we report first observations of SecDF protrusions from a fluid lipid bilayer, both in the presence and absence of its core partner in the cytoplasmic membrane of *E. coli*, translocon SecYEG. The asymmetric structure of SecDF along with a SecD mutant lacking the periplasmic P1 domain allowed orientational assignments. Experimental data were compared with simulated AFM images of the cytoplasmic and periplasmic faces of SecDF based on orientation of proteins in membrane data (30). The coassembly of SecDF with SecYEG into liposomes, denoted as SecYEG·DF, induced significant conformational changes. For example, SecYEG·DF exhibited a pronounced population of periplasmic protrusions >36 Å above the bilayer surface that was largely absent without the translocon. Distinctions in conformational dynamics were also observed between the coassembled system and SecDF alone, further suggestive of a direct SecYEG–SecDF interaction.

## Results

AFM provides visualization of membrane-external conformations and conformational dynamics of SecDF in near-native supported lipid bilayers. Following previous work with the Sec system, proteins were reconstituted into liposomes comprised of *E. coli* polar lipid and adsorbed onto freshly cleaved mica surfaces for AFM (27, 28, 29, 31). Prior to imaging, biochemical assays were performed to verify that the reconstituted proteoliposomes maintained the ability to translocate precursor proteins (Fig. S1) (32). Tapping mode in fluid imaging was applied to the supported lipid bilayers in aqueous buffer (10 mM Hepes [pH 7.6], 30 mM KCl, 1 mM MgCl_2_, 1 mM Tris(2-carboxyethyl)phosphine). The presence of a lipid bilayer was confirmed by observation of the characteristic 40 Å thickness (Fig. S2) (33). Topographical information, including the height and volume of individual SecDF protrusions above the membrane surface, was extracted algorithmically for statistical analysis (34).

### Orientation of SecDF in supported lipid bilayers

**Figure 1:**
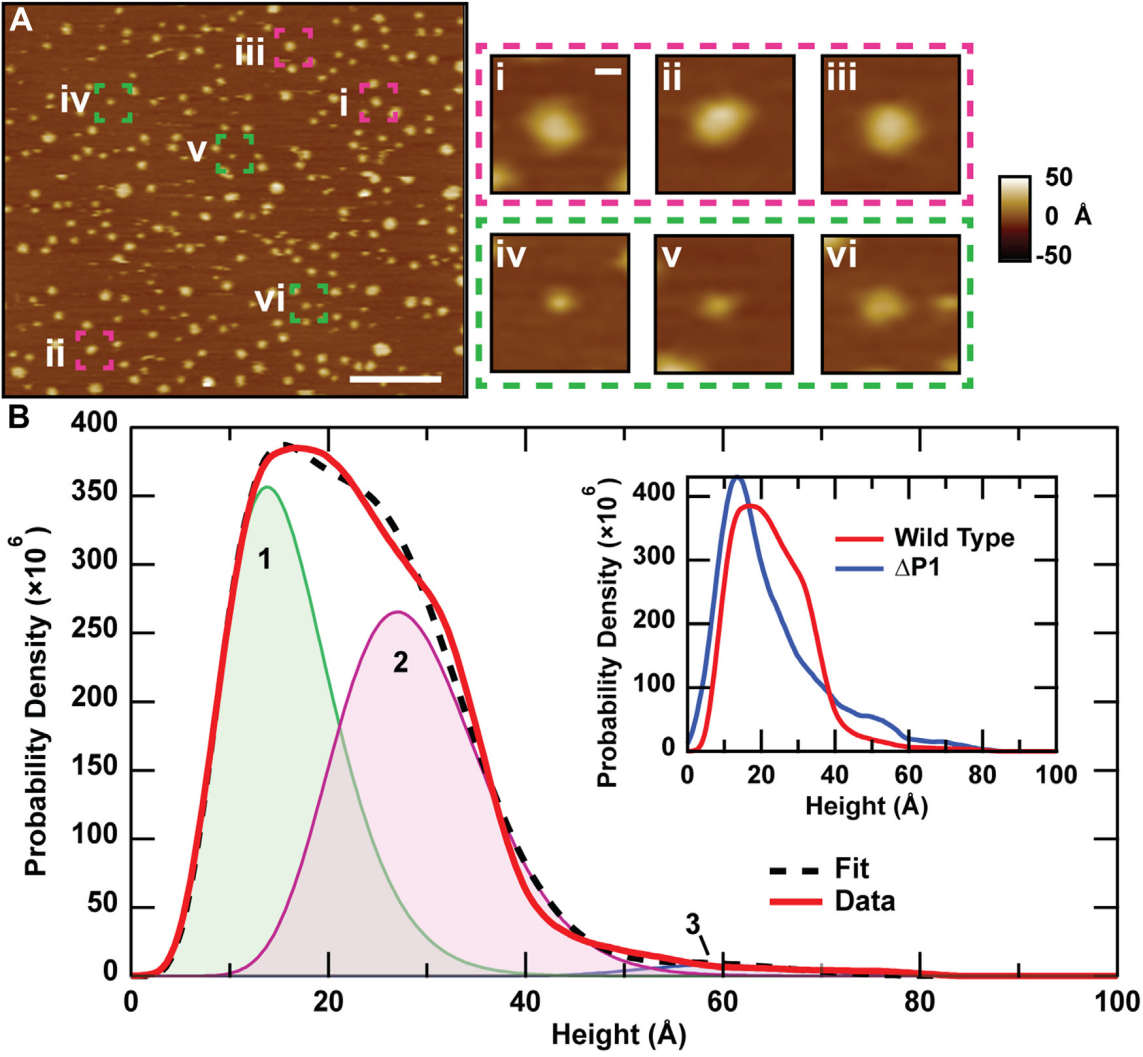
**Imaging SecDF protrusions reveals a multimodal height distribution.***A*, representative AFM image of SecDF in a supported lipid bilayer. Lateral scale bar represents 200 nm. Individual SecDF protrusions are identified by height as either the periplasmic (*magenta*, *i*–*iii*) or cytoplasmic (*green*, *iv*–*vi*) side of the protein. Lateral scale bar represents 10 nm. *B*, smoothed histogram of maximum SecDF height above the bilayer surface (*N* = 15,957 individual protrusions). Bayesian information criterion was applied to fit the data; the analysis required three model distributions with weights, *W*_1_ = 0.49, *W*_2_ = 0.49, and *W*_3_ = 0.02, respectively. The following are the peaks of each population: peak 1, 14 Å (*green*); peak 2, 27 Å (*magenta*); and peak 3, 60 Å (*blue*). *Inset,* comparison of histogram profiles for wildtype SecDF and SecDF lacking the periplasmic domain P1 (ΔP1). AFM, atomic force microscopy.

Proteoliposomes containing SecDF displayed numerous punctate features of varying heights (Fig. 1). Observing both faces of SecDF (*i.e.*, periplasmic and cytoplasmic protrusions) is expected as the sample preparation method scrambles the protein orientation in the bilayer (31). Structures have indicated that SecDF exhibits a topographic asymmetry between the periplasmic domain, which can protrude up to a maximum height of >60 Å above the bilayer and the cytoplasmic protrusion, which is much shorter (∼15 Å) (13, 22). Over 15,000 individual SecDF protrusions (features) were analyzed, and the results were plotted in a smoothed height histogram (Fig. 1*B*). Bayesian information criterion (BIC) was used to determine the optimal number of model distributions needed to fit the data without overfitting (Fig. S3) (35). Three model distributions were required with the first two having significantly greater weight (49% each) than the last. Gamma distributions were employed to capture the asymmetry of the data. The first two peaks in the height distribution fits occurred at approximately 14 and 27 Å, respectively, whereas the third and least populated peak (2% of total) corresponded to a protrusion height of about 60 Å. Comparing peaks in the AFM distribution to static crystal structure data can enable orientation and conformation assignments. In particular, simulated AFM images based on the crystal structure of the super-F form of SecDF (*Thermus thermophilus*; Protein Data Bank [PDB] ID: 5YHF) yields static protrusion heights of 16 and 36 Å for the cytoplasmic and periplasmic sides of the membrane protein complex, respectively (Fig. S4) (30). Analysis of the protrusions emanating from the F form (*T. thermophilus*; PDB ID: 3AQP) and I form (*Deinococcus radiodurans*; PDB ID: 5XAP) yields periplasmic heights of 47 and 63 Å, respectively. The cytoplasmic protrusion heights are similar; for all forms evaluated, they are ∼15 Å (Fig. S4). Based on these metrics, we assign peak 1 in the experimental distribution (Fig. 1*B*, *green*) to the cytoplasmic face of SecDF (agrees to within 10% of the average of the simulated cytoplasmic side data). The 27 Å height of peak 2 (Fig. 1*B*, *magenta*) appears too large to represent the cytoplasmic protrusion, but it is ∼9 Å smaller than the most compact super-F form of the periplasmic face. We employed a SecDF mutant lacking the P1 domain to aid in assignment of this peak (Fig. 1*B*, *inset*, ΔP1) (36). We see that the ΔP1 distribution takes on a different form, in which peak 2 in the wildtype distribution has been greatly suppressed. Quantitatively, upon BIC fitting (Fig. S5), only two gamma distributions were required to fit the ΔP1 data, and the weight of the subpopulation assigned to peak 2 was reduced significantly (greater than twofold). Hence, protrusions exhibiting heights ∼27 Å can be attributed to the P1 domain of SecD. Dynamics measurements (discussed later) revealed that this same population is conformationally stable, indicative of a resting state. The height of the least populated state (peak 3 in Fig. 1*B*) closely resembles the I-form of SecDF (agrees to within 5%). We note that aggregates were rare (<5% of total) and excluded from analysis by implementing a maximum height cutoff of 100 Å for all detected particles.

### The presence of SecYEG induces conformational shifts in SecDF

**Figure 2:**
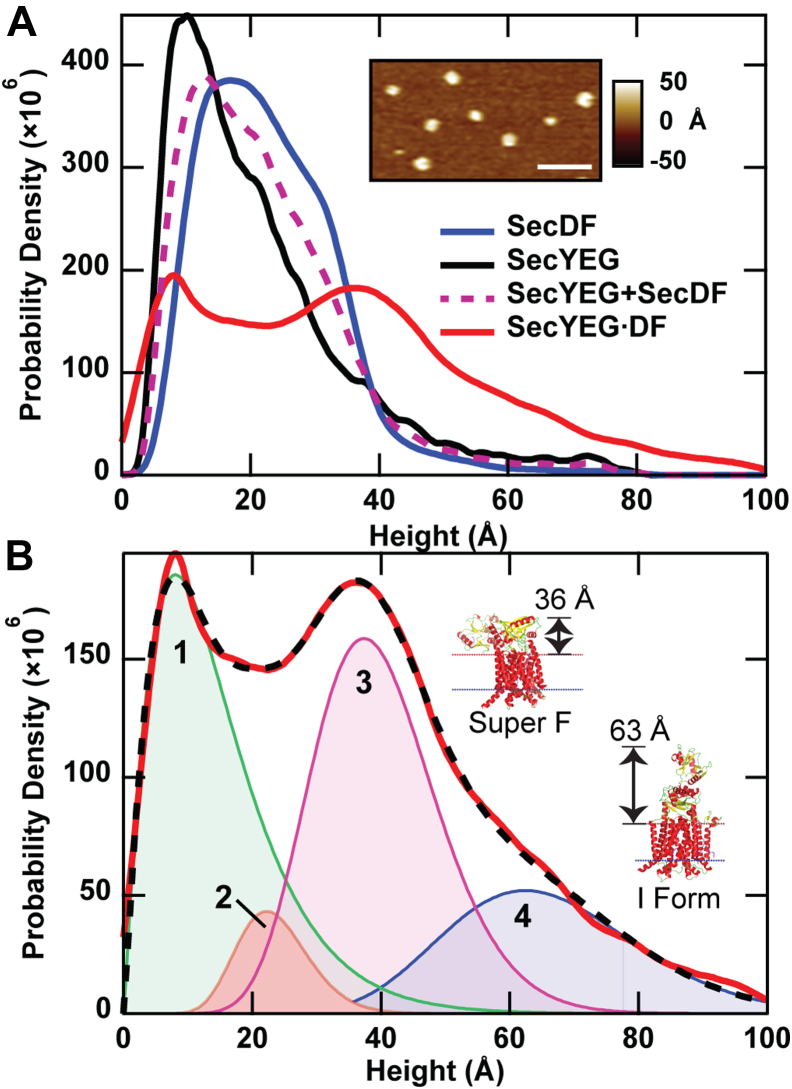
**Interaction with translocon SecYEG shifts SecDF conformation.***A*, height histograms for SecYEG alone (*N* = 3113; *black*), SecDF alone (*N* = 15,957; *blue*), normalized summation of their isolated distributions (SecYEG + SecDF; *purple dashed*), and the coassembled SecYEG·DF sample (*N* = 10,118; *red*). *Inset,* AFM image showing several SecYEG·DF protrusions. Lateral scale bar represents 100 nm. *B*, fitting the SecYEG·DF distribution prescribed four model distributions. The peaks and weights of each follows: peak 1: 9 Å, 37%; peak 2: 22 Å, 6%; peak 3: 37 Å, 38%; and peak 4: 62 Å, 19%. *Insets,* crystal structures of SecDF in the super-F (Protein Data Bank ID: 5YHF) and I form (Protein Data Bank ID: 5XAP) highlight conformational changes of the P1-head. The protrusion heights above the periplasmic side of the bilayer are indicated. AFM, atomic force microscopy; SecYEG·DF, co-assembled proteoliposomes containing SecYEG and SecDF.

As a central component of the holotranslocon, SecDF associates with SecYEG along with other components (5). Hence, to probe conformational changes of SecDF in a translocation-component environment, we applied AFM to proteoliposomes coassembled with SecYEG and SecDF (SecYEG·DF). The height distribution of SecYEG·DF protrusions (Fig. 2*A*, *red*) was much broader than that of SecDF alone (Fig. 2*A*, *blue*). It exhibited a substantial population at a height range of ∼60 Å, corresponding closely to I form and indicative of a highly dynamic macromolecular complex. The height distributions of proteoliposomes containing just SecYEG are also plotted for comparison (Fig. 2*A*, *black*; Fig. S6). Interestingly, the height distribution of the coassembled SecYEG·DF sample is significantly broader than the summation of the two height distributions corresponding to SecYEG and SecDF in isolation. The emergence of new topographic populations evinces an interaction between translocon SecYEG and SecDF.

Fitting the SecYEG·DF height distribution using BIC required four gamma distributions (Fig. 2*B*), as opposed to three in the case of SecDF in isolation (Fig. 1*B*). A prominent population in the SecYEG·DF height distribution (Fig. 2*B*, peak 3) is observed at 37 Å. This protrusion height is in very good agreement with simulations of the super-F form (agrees to within 3%). The weight of the SecYEG·DF height population at approximately 62 Å (Fig. 2*B*, peak 4), which was 2% when probing SecDF in isolation (Fig. 1*B*, peak 3) and is in very good agreement with crystal structure data of SecDF in the I form, has now increased greater than ninefold in the presence of the translocon SecYEG. As expected, analysis of SecYEG·DFΔP1 (*i.e.*, SecD with P1 deleted) indicates that the peaks at ∼37 and ∼62 Å correspond to the periplasmic side of SecD (Fig. S5). The two SecYEG·DF populations exhibiting the smallest heights (Fig. 2*B*, peaks 2 and 1, at ∼22 and ∼9 Å) are likely to correspond to the cytoplasmic side of SecDF (peak 2), which is not thought to interact directly with precursor proteins, the cytoplasmic side of SecYEG (peak 2), and to the periplasmic side of SecYEG (peak 1) (27). In addition to the crystal structures of SecDF, we simulated AFM images of SecYEG·DF based on a cryo-EM structure of the bacterial holotranslocon (*E. coli*; PDB ID: 5MG3). Analysis of the simulated periplasmic side image (Fig. S7) yielded a height of 37 Å, which is similar to the super-F form of SecDF from the crystal structure and is in excellent agreement with the AFM measured height peak at 37 Å.

### Analysis of protrusion volume

**Figure 3:**
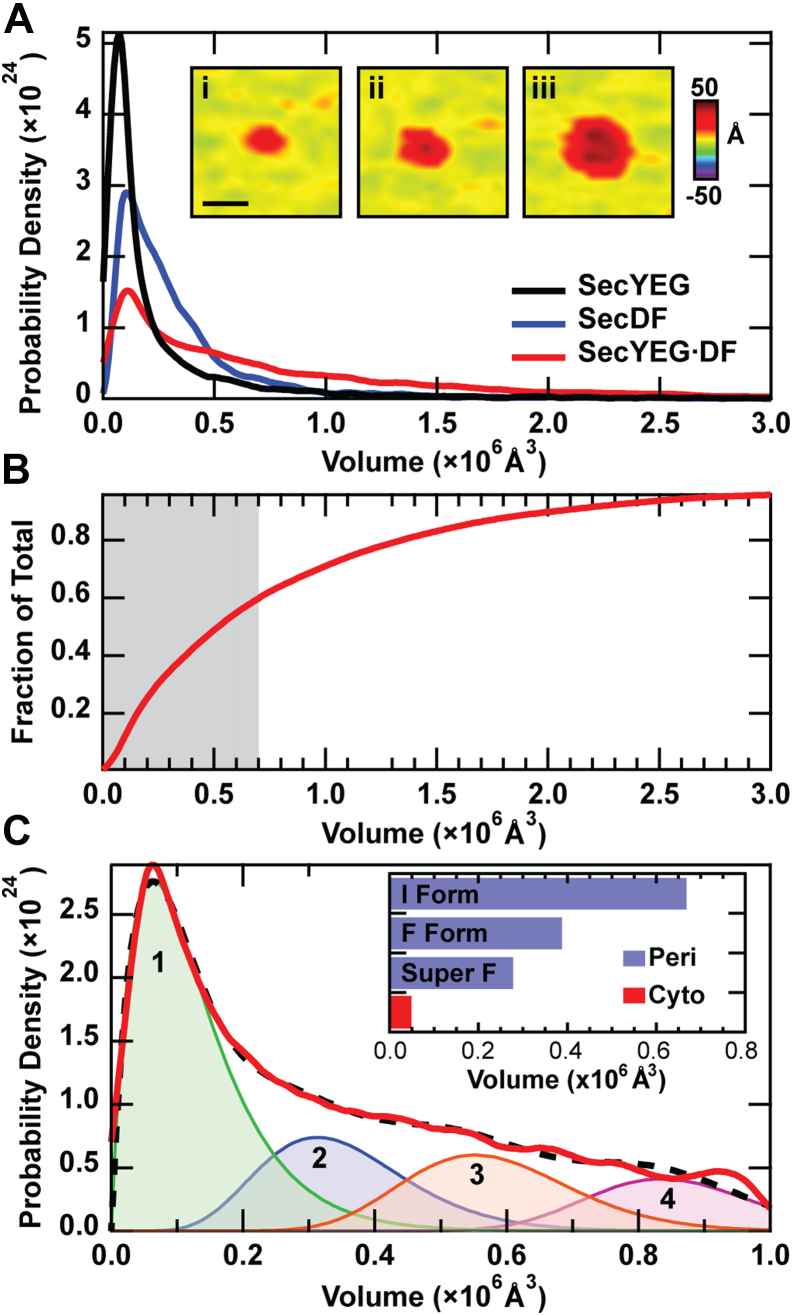
**Volume analysis of protein protrusions.***A*, volume histograms for three species of proteoliposomes: SecYEG alone, SecDF alone, and SecYEG·DF. *Insets* show representative images of SecYEG·DF protrusions exhibiting volumes: *i* = 0.27 × 10^6^ Å^3^, *ii* = 0.9 × 10^6^ Å^3^, and *iii* = 2.4 × 10^6^ Å^3^. Lateral scale bar represents 20 nm. *B*, the accumulated fraction of all features is shown. *C*, truncated volume histogram of SecYEG·DF representing all features with volumes up to 1.2 × 10^6^ Å^3^. The peak and weight of each gamma distribution of the fit (*dashed*) are provided: peak 1: 0.06 × 10^6^ Å^3^, 48%; peak 2: 0.31 × 10^6^ Å^3^, 20%; peak 3: 0.55 × 10^6^ Å^3^, 19%; and peak 4: 0.84 × 10^6^ Å^3^, 13%. *Inset,* simulated volumes for periplasmic protrusions of three SecDF conformations. The average cytoplasmic volume is also shown. SecYEG·DF, co-assembled proteoliposomes containing SecYEG and SecDF.

We also determined the protrusion volumes corresponding to SecDF in isolation, SecYEG in isolation, and SecYEG·DF (Fig. 3). All three of these samples exhibited prominent volume peaks in the monomeric range based on simulated AFM data (<0.7 × 10^6^ Å^3^, Fig. 3*C*, *inset*). This recapitulates previous work on SecYEG at similar protein concentration (27). The lightly populated high-volume tail of the SecYEG·DF distribution corresponds to a population of large lateral assemblies, an example image of which is shown (Fig. 3*A*, *inset iii*). As for the height distributions, when fitting the volumes *via* the BIC, SecYEG·DF required more model distributions then SecDF or SecYEG alone (Fig. S3). Approximately 60% of all SecYEG·DF features fall within the monomer range of SecDF (Fig. 3*B*, *gray shaded region*).

Simulated AFM images provide insight into how the volume of SecDF protrusions is likely affected by the presence of the translocon. On the cytoplasmic side, simulated SecYEG·DF is fourfold greater in volume than that of SecDF in isolation (which has an average of ∼0.05 × 10^6^ Å^3^ over the three forms: Super F, F, and I). In contrast, on the periplasmic side, the volume of SecYEG·DF is quite similar to the volume of the super-F form of SecDF, both of which are ∼0.2 × 10^6^ Å^3^; this could be a result of the SecD P1-head being positioned over the SecYEG exit site, as has been proposed (17). The periplasmic side of SecDF I form exhibits the largest simulated volume, 0.7 × 10^6^ Å^3^ (Fig. 3*C*, *inset*). To allow for detailed fitting, the small population of large volume features (>1.2 × 10^6^ Å^3^) was omitted, and the resulting BIC prescribed four model distributions, as indicated (Fig. 3*C*). Peaks 1 and 2 and most of peak 3 fall within the expected range for monomeric SecYEG, SecDF, and SecYEG·DF. Peak 4, which is centered at a volume of 0.84 × 10^6^ Å^3^, is the least populated volume peak (weight = 13%). This high-volume population likely corresponds to a higher order oligomeric structure of unknown stoichiometry.

### Conformational dynamics on the 100 ms timescale

**Figure 4:**
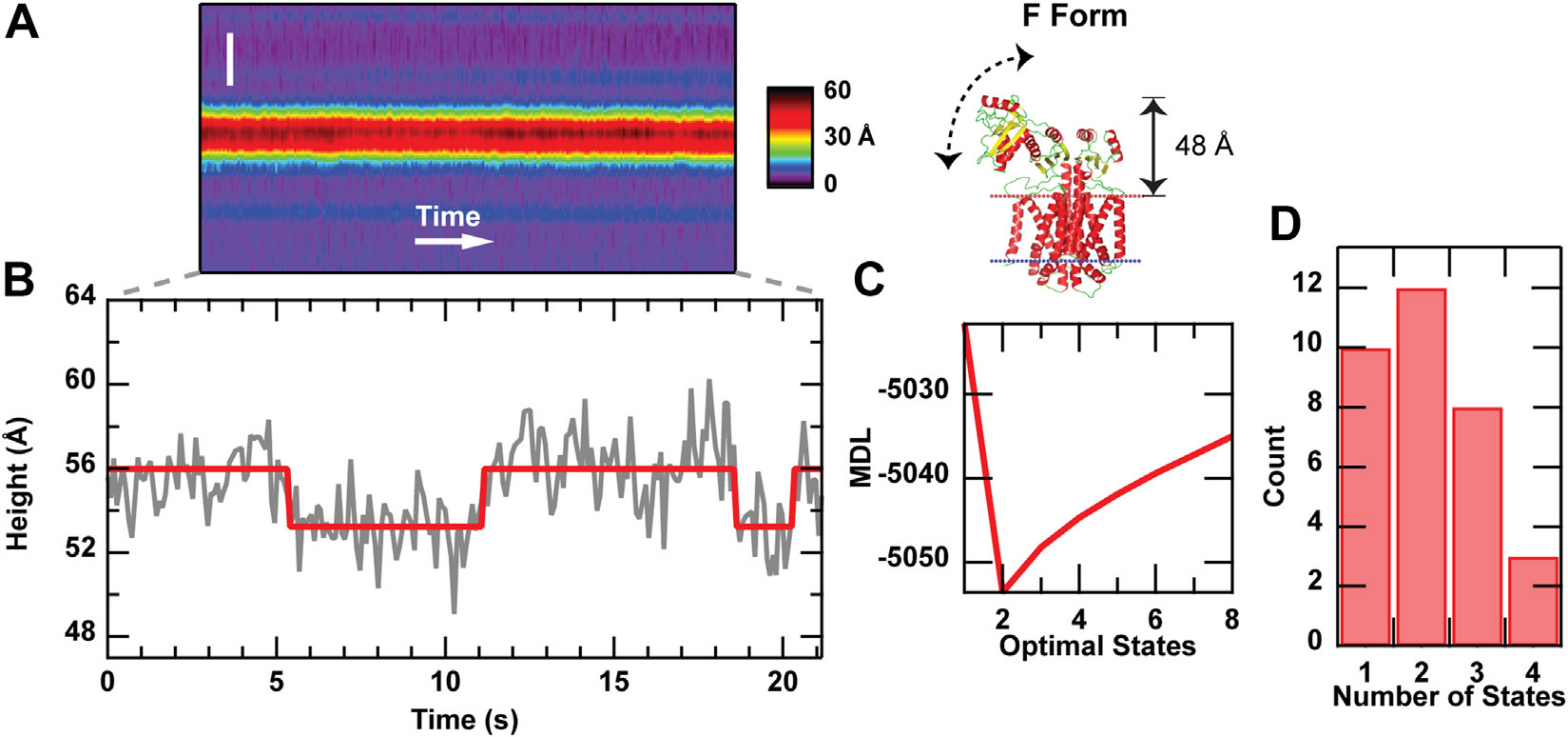
**Kymographs reveal conformational dynamics ofSecYEG·DF.***A*, kymograph of a single SecYEG·DF protrusion. The spatial axis is vertical (scale bar represents 50 nm), and time is horizontal (90 ms per line). The structure of the F form of SecDF (Protein Data Bank ID: 3AQP; including static protrusion height) is provided to highlight P1 motion. *B*, the maximum height (*gray line*) of the protrusion is extracted from the kymograph, and the output of the STaSI algorithm is overlaid (*red line*). *C*, plot of the minimum description length (MDL) used to determine the optimal number of states present. *D*, histogram of SecYEG·DF states (*N* = 33 kymographs). SecYEG·DF, co-assembled proteoliposomes containing SecYEG and SecDF.

Indicative of active macromolecules probed in fluid environments, the broad nature of the SecYEG·DF histogram is suggestive of significant conformational dynamics. Transiently occupied conformational states cause broad shoulders in height and volume distributions. Hence, we sought to directly observe conformational dynamics. To do so, we enhanced the temporal resolution to ∼100 ms by repeatedly scanning over the same individual periplasmic SecDF protrusion in one dimension, rather than in two dimensions. Analysis of the resulting kymographs (Fig. 4) revealed changes in the maximum height of the protrusion above the membrane as a function of time. We applied the Step Transition and State Identification (STaSI) algorithm to determine states and kinetics (37). This analysis applies Student's *t* test to the kymograph data and calculates the number of states present in the data using the minimum description length principle (Fig. 4, *C* and *D*) (37). The STaSI output is a piecewise function that identifies states present in the kymograph (Fig. 4*B*, *red trace*). The kymographs of SecYEG·DF (Table 1) exhibited up to four states, with two states being the most common (Fig. 4*D*). In contrast, analogous analysis of SecDF in the absence of SecYEG showed one state as the most common, the number of states histogram decreased monotonically thereafter (Fig. S8). The cytoplasmic protrusion of SecDF was also analyzed and found to be quiescent (Fig. S9), as expected.

**Table 1:** The presence of SecYEG enhances the conformational dynamics of SecDF

Conformational dynamics of SecYEG·DF *versus* SecDF		
Measurement	SecYEG·DF	SecDF
Kymographs	33	21
Peak number of states	2	1
Line scans	7064	4136
Total time (s)	636	372
Total transitions	119	30
Transition rate (s^−1^)	0.19	0.08

Comparison between SecDF in isolation (*right column*) and SecYEG·DF (*left*) shows an enhancement in the most likely number of states observed. In addition, a greater than twofold increase in the rate of transitions between states is apparent for the coassembled SecYEG·DF sample. Periplasmic side features were analyzed in all cases.

Kinetic information was determined from the kymograph data. For the periplasmic side of both SecDF and SecYEG·DF, the number of transitions between states was determined and used to calculate an average transition rate per second (Table 1). For SecYEG·DF, this rate was 0.19 1/s, whereas for SecDF alone, the rate was 0.08 1/s. Hence, the periplasmic protrusions of SecYEG·DF are approximately twofold more conformationally dynamic than that of SecDF in isolation. Fig. S9 shows analogous data for the cytoplasmic side of SecDF.

## Discussion

Traditional structural methods provide static snapshots outside the native environments. By imaging integral membrane proteins in fluid lipid bilayers, AFM data complement high-resolution structures. We focused on the dynamic membrane-external topography of SecDF and SecYEG·DF. Previous studies have implicated SecDF in a late step of protein translocation (8, 38), acting from the periplasmic side of the membrane, but mechanistic understanding remains elusive (39, 40). This motivates the need to develop approaches that can directly visualize translocation machinery in action.

Proteoliposome SecDF exhibited a height distribution with two prominent peaks, comprising 98% of the total. Roughly half of the SecDF population measured ∼14 Å above the bilayer surface (Fig. 1, peak 1, weight = 49%). This population is in good agreement with the expected height of the cytoplasmic SecDF protrusion (∼15 Å for all structures). When comparing the experimental distributions to simulated AFM images, the peak at ∼27 Å (Fig. 1, peak 2, weight = 49%) appears too high above the bilayer to be associated with the cytoplasmic face of the protein. Experiments with SecD lacking the P1 domain corroborated this notion. Our data indicate that the ∼27 Å population is the periplasmic face of SecDF in a highly compact conformation distinct from previously reported structures. The dynamic measurements further indicate that this conformation is highly stable, indicative of a SecDF resting state. A potential explanation for the lack of correlation with existing structures is that our AFM measurements are carried out in near-native *E. coli* polar lipid bilayers. Charged lipid head groups could interact with and attract the mobile P1 domain toward the bilayer surface, causing a conformation that is not evident in structural studies lacking a lipid bilayer membrane. A sample preparation/imaging artifact is also formally possible but unlikely because of the very good agreement between the high-resolution structures and AFM-measured conformations when SecYEG was incorporated, as shown in Fig. 2. In this case when combined with SecYEG, the P1 domain of SecD could become shielded from an underlying lipid interaction. The sparsely populated peak at ∼60 Å (Fig. 1, peak 3) is in good agreement with the I form of SecDF that is thought to be directly involved with precursor handling. However, this peak is very lightly populated (weight = 2%) when SecDF is in isolation. A majority of SecDF complexes do not appear to be in active conformations when probed in the absence of translocon SecYEG.

Our work sheds light on the conformational implications of interactions between SecDF and SecYEG, both of which are central components of the holotranslocon. Significant changes in conformation and conformational dynamics were observed when comparing isolated SecDF or isolated SecYEG to coassembled proteoliposomes containing SecYEG and SecDF together. These results are consistent with previous studies implicating chains in SecDF contacting SecY and SecG, giving rise to a functional state involved in precursor translocation across the membrane (15, 41). Indeed, if SecDF and SecYEG did not associate or interact, one would expect that the height distribution of coassembled SecYEG·DF would be similar to the summation of the two isolated-protein distributions. In contrast, the summation distribution (Fig. 2*A*, SecYEG + SecDF) is qualitatively and quantitatively distinct from that of SecYEG·DF. While the most populated peak in the SecYEG·DF distribution (Fig. 2*B*, peak 3) is in good agreement with the height of the super-F form (experimental value = 37 Å, simulated value = 36 Å), peak 4 is in good agreement with the I form (experimental value = 62 Å, simulated value = 63 Å) and is also well populated. We posit that peak 4 in the SecYEG·DF distribution (Fig. 2*B*, weight = 19%) corresponds to peak 3 of the SecDF alone data (Fig. 1*B*, weight = 2%). However, the weights differ significantly between the two samples. When translocon SecYEG is available to associate with SecDF, the periplasmic side of SecDF is greater than ninefold more likely to occupy an I-form-like conformation, which is hypothesized to be a translocation-active state.

Though SecYEG has been shown to acquire dimer and higher order quaternary structure (42, 43, 44), SecDF is functional as a monomer (14). Sec system protrusion volumes were used to deduce oligomeric state (27). As expected, most of the SecDF volume data fell within the monomeric volume range. The lightly populated higher volume populations of SecYEG·DF may correspond to higher order SecYEG structures, such as (SecYEG)_2_·DF. Further work will be required to verify stoichiometry assignment.

Kymographs visualized membrane-external conformational dynamics in real time (resolution ∼100 ms) in the absence of PMF and other factors including YidC and precursor protein. The SecYEG·DF data show distinct conformations interconverting (Fig. 4). Analogous analysis of SecDF in isolation resulted in an approximate twofold reduction in the observed transition rate between states. This implies that SecDF is less prone to change conformations when subject to thermal driving forces if it is not allowed to associate with the translocon. The changing kinetics were commensurate with changes in the state number distributions. In the kymograph data, periplasmic SecYEG·DF protrusions were most likely to be found in two distinct states (*i.e.*, heights above the membrane surface). In contrast, isolated SecDF was more stable and was most often found occupying a single conformational state. These data support the notion that SecDF adopts a stable resting state when it is in isolation but is conformationally activated by the presence of SecYEG.

We applied AFM imaging to the integral membrane translocation factor SecDF in physiologically relevant conditions. This single-molecule technique is a powerful complement to traditional methods that require proteins to be in a fixed conformation because of crystallization or cryopreservation. Our work highlights the flexibility of the periplasmic P1 domain, the motion of which has been implicated in polypeptide translocation, and suggests that SecDF and SecYEG can interact directly without YidC. In the future, it will be interesting to include additional components of the bacterial holotranslocon coupled with advanced methodologies (45, 46). For example, developing an assay that utilizes PMF and precursors could address questions in real time about the mechanics of protein secretion, such as identifying specific steps where PMF is utilized (36). High-precision single-molecule methods are poised to help unravel the asynchronous conformational gymnastics associated with protein translocation across membranes.

## Experimental procedures

### Protein purification

SecDF was purified from strain BL21(DE3) harboring a plasmid pEXP10 (original pET546 from Arnold Driessen) encoding *secF* with a His tag at the C terminus, *secD* and *yajC* (19). So that the data gathered would be consistent with future studies involving PMF, all solutions contained chloride, which in previous publications contained acetate. YajC was not detected in the final purified complex. SecYEG was purified from strain C43(DE3) (47) harboring a plasmid encoding *secE* with a His tag at the N terminus, *secYC329S*, *C385S*, and *secG* (48). Cells were broken by passage through a French pressure cell (8000 psi), and the membranes were isolated by centrifugation and solubilized in dodecyl-β-maltoside (DBM). SecDF and SecYEG were purified by chromatography using a HisTrap column (GE Healthcare). Samples were stored at −80 °C in 20 mm Tris–Cl, pH 8, 0.3 M NaCl, 10% glycerol, 2 mM DBM, and 2 mm DTT. Purification of SecD lacking the P1 domain (ΔP1) was carried out in the same manner as described previously for wildtype.

### Preparation of proteoliposomes

Following previous protocols (31), unilamellar liposomes were prepared by extrusion of *E. coli* polar lipids (Avanti), which were suspended in 10 mM Hepes at pH 7.6, 30 mM KCl, 1 mM MgCl_2,_ through membranes with a 100-nm pore diameter, using a Liposofast (Avestin). To form proteoliposomes, the liposomes were swelled but not disrupted, using a ratio of detergent to lipids of 4.65 mM DBM to 5 mM lipids (49). After swelling for 3 h at room temperature, the proteins to be incorporated were added with 3 μM each of the protease inhibitors pepstatin and leupeptin. For AFM studies of SecDF in isolation, input concentration was 7.3 μM SecDF; for coassembled SecYEG·DF, input was 14.6 μM SecYEG and 7.3 μM SecDF. Incubation was further continued for 1 h at room temperature, followed by addition of BioBeads SM-2 (Bio-Rad) to remove the detergent. The proteoliposomes were isolated by centrifugation at 436,000*g*, 20 min at 4 °C, in a TL100.1 rotor (Beckman). The pellet was suspended in the same solution and centrifuged again as earlier. The final pellet was suspended to give a concentration of ∼1.5 μM SecD (for isolated SecDF) or ∼3 μM SecY (for SecYEG·DF). Suspensions were stored at −80 °C.

### Biochemical activity assay

The precursor of outer membrane protein A (pOmpA), SecA, and SecB were prepared as described (50). Translocation of [^14^C] leucine pOmpA into proteoliposomes was carried out in glass tubes (12 by 75 mm) at 30 °C. The mixture contained 10 mM Hepes (pH 7.6), 50 mM KCl, 1 mM MgCl_2_, 2 mM MDTT, 1 mM EGTA, 400 μM ATP, and an ATP-regenerating system consisting of 7.5 mM phosphocreatine and 37 mg/ml creatine phosphokinase. SecB (1.2 μM tetramer), SecA (0.8 μM dimer), and proteoliposomes SecYEG·DF (0.5 μM SecYEG and 0.5 μM SecDF) were added to the mixture, followed by the addition of radiolabeled precursor. The level of radioactivity (counts per minute per microliter) in each mixture was determined by removing 5 μl (in duplicates) into 100 μl water held in scintillation vials to which 3 ml of 30% ScintiSafe (Thermo Fisher Scientific) was added. The concentration of precursor (in micrograms per milliliter) in each mixture was determined from the specific activity (counts per minute per microgram) of purified proteins. The reaction was initiated by transferring the glass tube to a water bath at 30 °C. At each time step, 8 μl of the reaction mixture were added to tubes held on ice containing 6 μl of 42 mM EDTA to stop ATP hydrolysis, 25 mM DTT, and 26.7 mM urea. Proteinase K (5 units/ml, 15 min on ice) was added to degrade untranslocated proteins, and digestion was terminated by trichloroacetic acid precipitation. The washed precipitate was dissolved in gel sample buffer containing DTT (10 mM) for analysis by electrophoresis. The radioactivity in the protein bands in the gels of the translocation assays was measured using a Fujifilm FLA 3000 phosphorimager in the linear range of its response, and the molarity of the full-length protected precursors was estimated by comparison with samples taken from the same reaction mix, which had not been subjected to proteinase K digestion but had been applied to the same gel. Analyses of the translocation data were carried out as described (50). Origin software (OriginLab Corporation) was used to fit data to a single exponential rise to maximum with the equation $y=y_{o}+A\left(1-{\text{e}}^{-k\left(t-t_{o}\right)}\right)$, where *A* is the maximal amplitude of the reaction, *k* is the apparent rate constant, and *t*–*t*_0_ corrects for the initial time lag. The time for the reaction mixture to come to 30 °C was measured using a thermocouple, and it was found to be between 15 and 30 s depending on the volume.

### Sample preparation for AFM

AFM samples were diluted to have a SecDF concentration of 70 nM in imaging buffer (10 mM Hepes [pH 7.6], 30 mM KCl, 1 mM MgCl_2_, 1 mM (Tris(2-carboxyethyl)phosphine). About 100 μl of the diluted solution was deposited onto a freshly cleaved mica surface. For proteoliposomes containing SecYEG and DF (SecYEG·DF), a SecYEG concentration of 140 nM was used. Samples were incubated on ice for 45 min, allowing the proteoliposomes to rupture and form supported lipid bilayers, which are suitable for AFM imaging. After incubation, samples were rinsed five times with 100 μl of the imaging buffer to remove loosely bound material.

### AFM

AFM images were taken at ∼32 ˚C in tapping mode using a commercial instrument (Asylum Research Cypher) in imaging buffer (defined previously). Tips (Model AC40; Bruker) with nominal spring constants of ∼0.1 N/m were used. A tip-sample force of <100 pN was maintained, which was estimated by comparing the free amplitude to the set point amplitude. The following image parameters were used: 256 × 256 pixels; scan size 1 μm × 1 μm; and scan speed 6 μm/s. Kymograph parameters were as follows: 1-D pixel size 1.9 nm and scan speed 14 μm/s. To minimize the effects of drift, the temporal duration of each kymograph was set to 10 to 20 s.

### AFM image analysis

As is common with AFM analysis, images were flattened (second order) to minimize background tilt. Individual particles were extracted in each image using the Hessian Blob Algorithm, implemented with custom software (Igor Pro 7; WaveMetrics) (34). A local background level was determined for each individual molecule *via* Laplace interpolation, providing an accurate baseline from which various metrics were measured. Each particle’s height was determined from the highest pixel above the background level of the molecule. Smoothed height and volume histograms (probability density functions) were generated using an Epanechnikov kernel (Igor Pro 7) and were normalized to unity area with the abscissa expressed in SI units (*i.e.*, m or m^3^). To determine the number of subpopulations and avoid overfitting, BIC was implemented using gamma model distributions (35, 51).

### Kymograph analysis

For analyses of kymographs, a modification was made to the Hessian Blob Algorithm that can be applied to linear features (Fig. S10) (52). In this method, rather than minimizing the determinant of the Hessian matrix, the image skeleton is extracted. The lateral boundaries, central backbone, and maximum height pixel for each line within the kymograph are extracted. Traces of maximum height *versus* time were analyzed by the STaSI state detection algorithm (37, 53) implemented in MATLAB (MATLAB, MathWorks). Briefly, the algorithm detects step transitions using Student’s *t* test before being grouped into segments *via* hierarchical clusters. An optimal number of states was determined for each kymograph by considering the simplest model, which gives the least fitting error (minimum description length) (54). Kinetics were deduced by counting transitions between states.

### Simulation of AFM images

Simulated AFM images were constructed using custom software (Igor Pro 7) as described (55). Briefly, we computed the morphological dilation between three-dimensional protein models (high-resolution structures) and an AFM tip. In the structures, each atom was modeled as a sphere with van der Waals radius (56). The AFM tip was modeled as a cone (angle = 17.5°) with a tip comprising a nested inner and outer sphere, *R*_inner_ = 4 nm < *R*_outer_ = 8 nm with a spherical overlap cross section of 2.8 nm.

## Data availability

All data needed to evaluate the conclusions in the article are present in the article and/or the supporting information section. Additional data related to this article may be requested from the authors.

## Supporting information

This article contains supporting information.

## Conflict of interest

The authors declare that they have no conflicts of interest with the contents of this article.
