# Supplementary material for: The conformations and basal conformational dynamics of translocation factor SecDF vary with translocon SecYEG interaction
Source: J Biol Chem. 2022 Aug 22;298(10):102412. doi: 10.1016/j.jbc.2022.102412 (PMC9508474; doi:10.1016/j.jbc.2022.102412)
Supplement: Supplementary Information [file mmc1.pdf]

Supplementary Information

The conformations and basal conformational dynamics of translocation factor SecDF vary with translocon SecYEG interaction

D.R. Weaver[1], D.N. Amin[2], and G.M. King[1,2,*]

[1]Department of Physics and Astronomy, University of Missouri, Columbia, MO 65211, USA

[2]Department of Biochemistry, University of Missouri, Columbia, MO 65211, USA

[*]Corresponding Author: kinggm@missouri.edu

Table of Contents

Page 1 ….. Fig. S1. Translocation activity assay.

Page 2 ….. Fig. S2. Verification of lipid bilayer.

Page 3 ….. Fig. S3. Bayesian Information Criterion (BIC) analysis.

Page 4 ….. Fig. S4. Simulated AFM images reveal protrusion shape and approximate height above the bilayer.

Page 5 ….. Fig. S5. Analysis of SecD lacking the P1 domain.

Page 6 ….. Fig. S6. Representative AFM data.

Page 7 ….. Fig. S7. Simulated AFM images of SecYEG·DF.

Page 8 ….. Fig. S8. SecDF in isolation exhibits minimal conformational dynamics in the periplasm.

Page 9 ….. Fig. S9. The cytoplasmic side of SecDF is conformationally quiescent.

Page 10 ….. Fig. S10. Flow chart of the line detection algorithm.

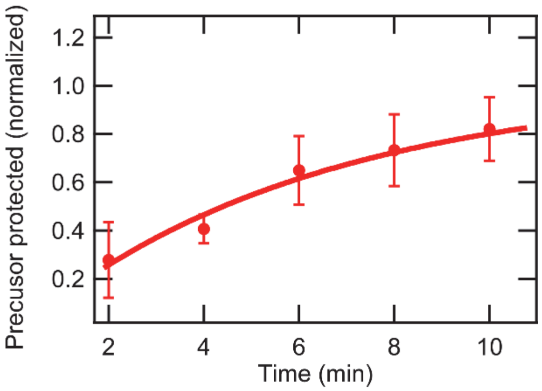

Fig. S1. Translocation activity assay. The translocation of radioactive precursor of outer membrane protein A into proteoliposomes containing SecYEG, SecDF, and SecA was quantified over time. A fit (solid line) to the experimental data ($N = 3$, standard deviations are shown) yields an apparent rate constant, $k = 0.16\ \text{min}^{-1}$. Control experiments carried out in the absence of ATP resulted in a mean value of 0.04 precursor protected after the full time course (10 min).

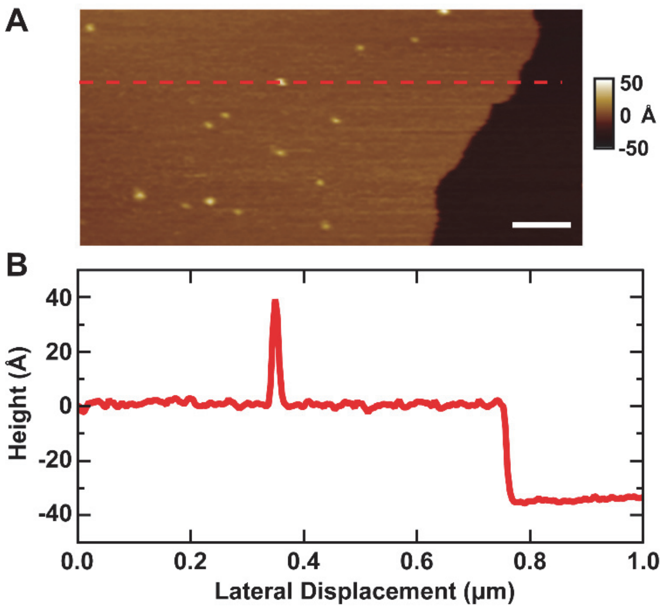

Fig. S2. Verification of lipid bilayer. (**A**) AFM image exhibiting two surfaces of different heights, one corresponding to the upper leaflet of the supported lipid bilayer (light brown) and the other (dark brown) to the supporting mica surface. Lateral scale bar is 100 nm. (**B**) Measured height across the dashed line drawn in (A), where the characteristic ~40 Å thickness of the lipid bilayer is confirmed. A punctate membrane protein protrusion is also observed in the line scan.

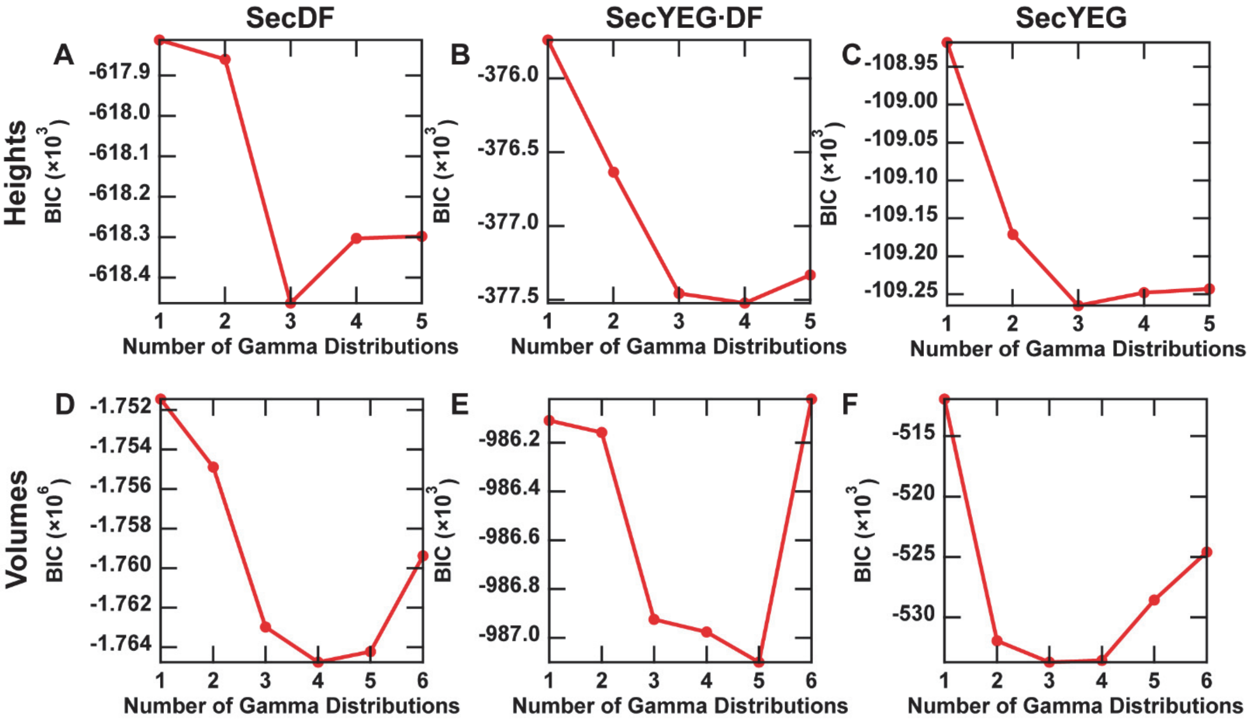

Fig. S3. Bayesian Information Criterion (BIC) analysis. BIC is applied to histograms of heights (**A-C**) and volumes (**D-F**) of the samples indicated. The analyses determine the optimal number of model distributions without overfitting. We note that broad distributions, indicative of conformational dynamics of active biological macromolecules, often require several model distributions to optimally fit.

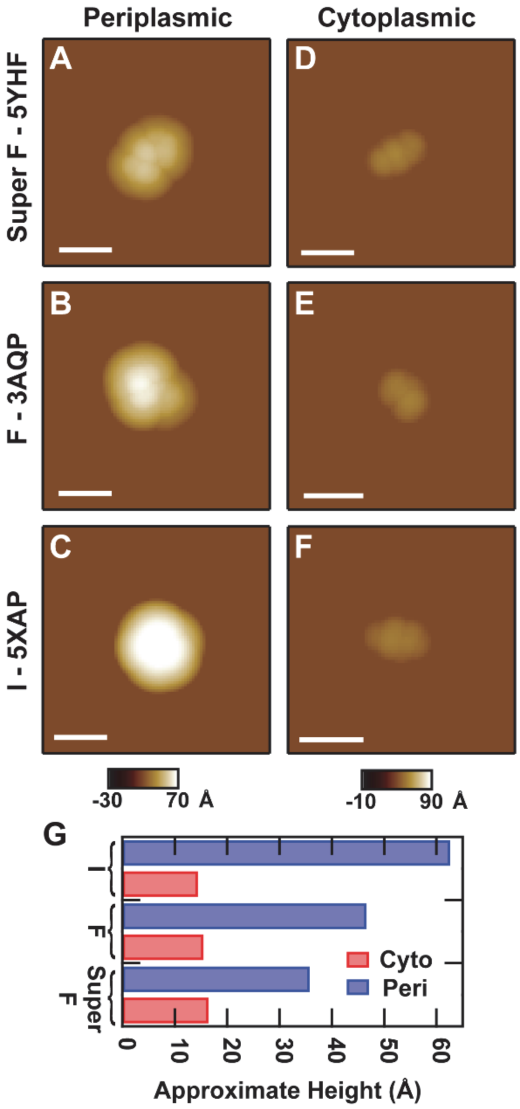

Fig. S4. Simulated AFM images reveal protrusion shape and approximate height above the bilayer. (**A**)-(**F**) Simulated images of the periplasmic (left column) and cytoplasmic (right column) side of SecDF protruding from a lipid bilayer from three crystal structures corresponding to Super F form (PDB: 5YHF), F form (3AQP), and I form (5XAP). Lipid bilayer positioning and vertical offsets based on OPM data base: https://opm.phar.umich.edu/. Lateral scale bar is 10 nm, pixel spacing is 5 Å. (**G**) Comparison of the height of each simulated structure calculated as the difference between the maximum and minimum pixels in the image.

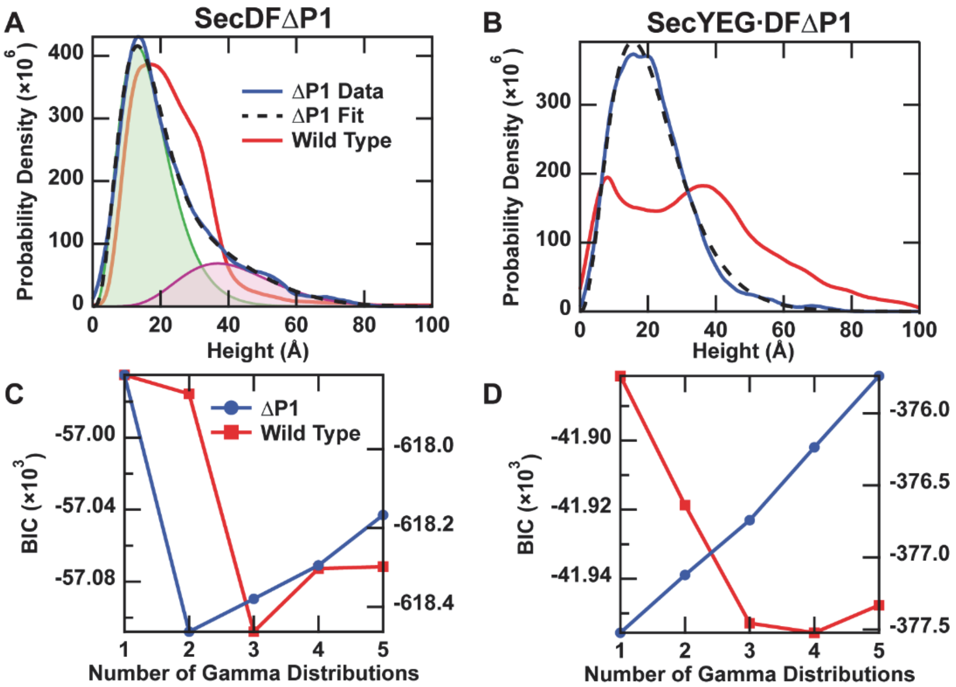

Fig. S5. Analysis of SecD lacking the P1 domain. BIC is applied to membrane external protrusions of SecDF mutants for (**A**), (**C**) SecDFΔP1 in isolation (*N*=1,490) and (**B**), (**D**) co-assembled with the translocon (SecYEG·DFΔP1, *N*=1,088). Each mutant histogram is overlaid on the wild-type data for comparison. In both cases, the optimal number of Gamma distributions needed to fit the mutant data is less than the wild-type, indicating a reduction in the number of conformational states.

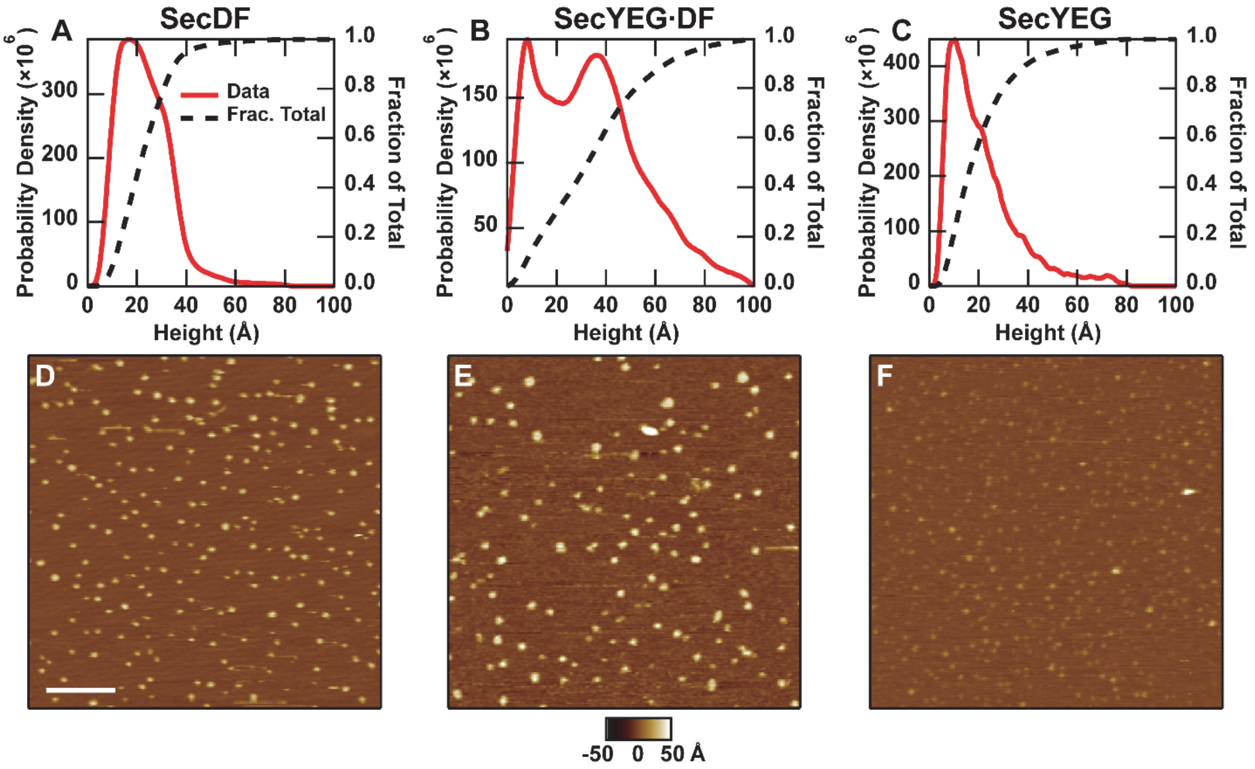

Fig. S6. Representative AFM data. Height histograms and representative AFM images of SecDF (**A** & **D**), SecYEG·DF (**B** & **E**), and SecYEG (**C** & **F**). The accumulated fraction of total features is also shown (black dashed traces). The false color vertical scale on all three images is the same and indicated; the lateral scale bar is 200 nm and applies to all images as well.

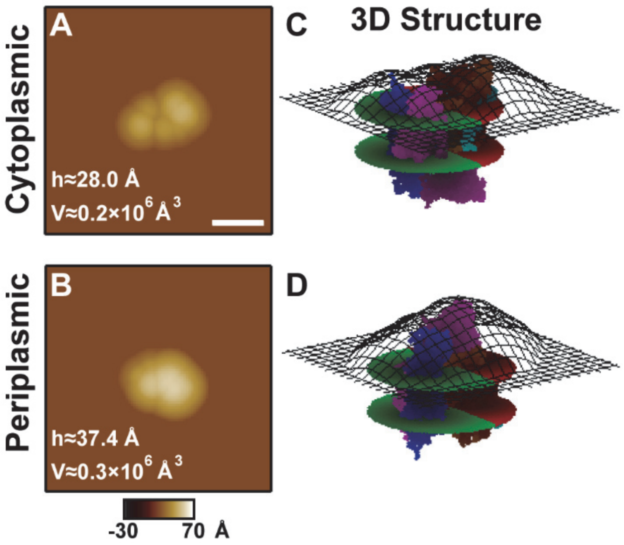

Fig. S7. Simulated AFM images of SecYEG·DF. Simulated AFM images were generated from the cryo-EM structure with SecDF in a membrane-facing conformation (*Escherichia coli*– PDB 5MG3 with YidC deleted). (**A**), (**B**) Simulated AFM images of the periplasmic and cytoplasmic domains of SecDF, respectively. Lateral scale bar is 10 nm, pixel spacing is 5 Å. (**C** & **D**) Wire mesh surface plots illustrate tip convolution on both sides of the bilayer. The following color code was used: purple (SecD), blue (SecF), brown (SecY), teal (SecE). Note that SecG is not visible in this orientation.

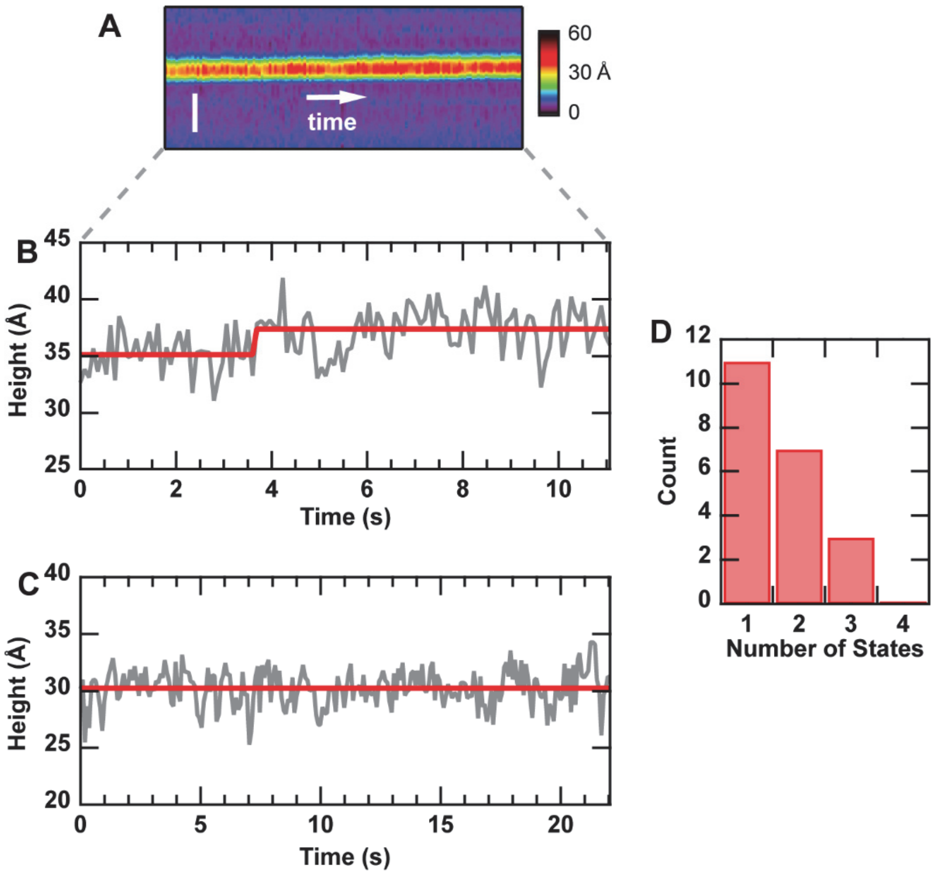

Fig. S8. SecDF in isolation exhibits minimal conformational dynamics in the periplasm. (**A**) Kymograph of an individual periplasmic SecDF protrusion. The scale bar is 50 nm, time is plotted on the horizontal axis (90 ms per line). (**B**) The maximum height (gray line) of the protrusion is extracted from the kymograph and the output of the STaSI algorithm is overlaid (red line). (**C**) Representative data from a different protrusion exhibiting no transitions (one state). (**D**) Histogram of periplasmic SecDF states (*N*=21 kymographs).

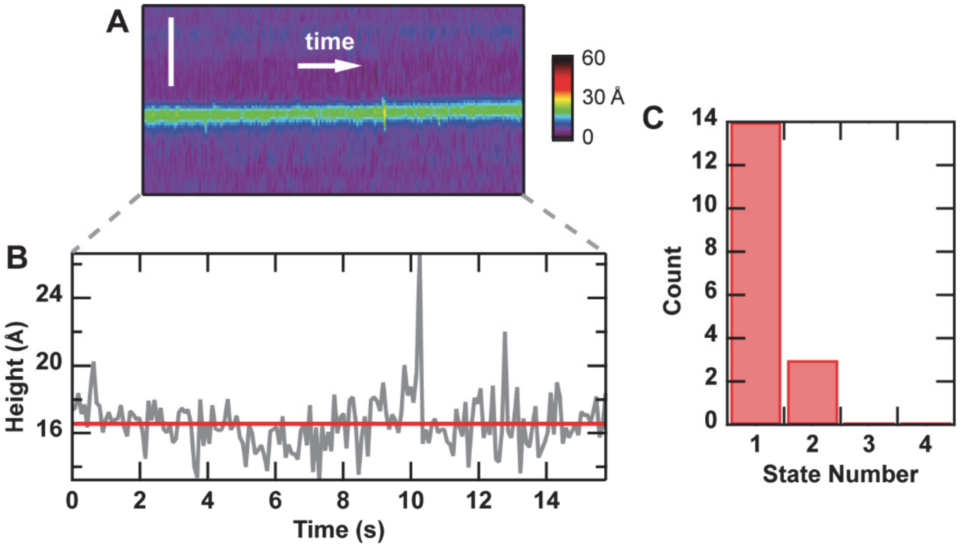

D

	SecDF-Cyto
Kymographs	17
Peak Number of States	**1**
Line Scans	2644
Total Time (s)	238
Total Transitions	5
Transition Rate (s^{-1})	**0.02**

Fig S9: The cytoplasmic side of SecDF is conformationally quiescent. (**A**) Kymograph of an individual cytoplasmic SecDF protrusion. The scale bar is 50 nm, time is plotted on the horizontal axis (90 ms per line). (**B**) The maximum height (gray line) of the protrusion is extracted from the kymograph and the output of the STaSI algorithm is overlaid (red line). (**C**) Histogram of cytoplasmic SecDF states (*N*=17 kymographs). (**D**) Kinetic information extracted across all kymographs.

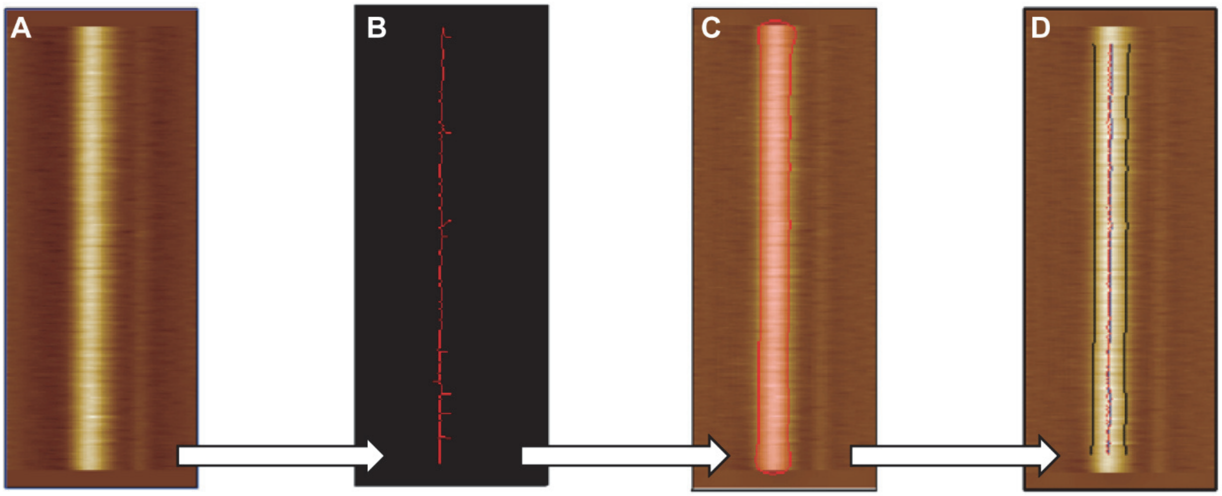

Fig. S10. Flow chart of the line detection algorithm. (**A**) The raw kymograph is padded on the top and bottom so it can be extracted as a particle. (**B**) Skeleton of the padded kymograph. (**C**) The skeleton is filled outward until the concavity changes. Finally, (**D**) the top and bottom caps are removed to obtain the lateral boundaries (black), central backbone (blue), and maximum heights (red).
